# Tertiary Lymphoid Structures as Mediators of Immunotherapy Response

**DOI:** 10.3390/cancers14153748

**Published:** 2022-08-01

**Authors:** Raj G. Vaghjiani, Joseph J. Skitzki

**Affiliations:** 1 Department of Surgical Oncology, Roswell Park Comprehensive Cancer Center, Buffalo, NY 14263, USA; raj.vaghjiani@roswellpark.org; 2 Department of Immunology, Roswell Park Comprehensive Cancer Center, Buffalo, NY 14263, USA

**Keywords:** tertiary lymphoid structures, HEV-like vessels, tumor immunology, cancer immunotherapy

## Abstract

**Simple Summary:**

Tertiary lymphoid structures (TLS) are anatomic entities that are similar to, but distinct from, secondary lymphoid structures (e.g., lymph nodes) that allow for a host’s own immune system to respond in a more targeted and efficacious way. TLS are increasingly recognized as markers of prognosis in cancer patients and are now being implicated as direct mediators of immunotherapy efficacy. The inherent properties of TLS, as well as their cellular constituents, are being elucidated across tumor types, with commonalities becoming more apparent. Given the importance of TLS in a patient’s response to malignancy, the ability to induce TLS promises to be an advantageous therapeutic avenue and already appears feasible in preclinical models.

**Abstract:**

Since its first application in the treatment of cancer during the 1800s, immunotherapy has more recently become the leading edge of novel treatment strategies. Even though the efficacy of these agents can at times be predicted by more traditional metrics and biomarkers, often patient responses are variable. TLS are distinct immunologic structures that have been identified on pathologic review of various malignancies and are emerging as important determinants of patient outcome. Their presence, location, composition, and maturity are critically important in a host’s response to malignancy. Because of their unique immunogenic niche, they are also prime candidates, not only to predict and measure the efficacy of immunotherapy agents, but also to be potentially inducible gatekeepers to increase therapeutic efficacy. Herein, we review the mechanistic underpinnings of TLS formation, the data on its relationship to various malignancies, and the emerging evidence for the role of TLS in immunotherapy function.

## 1. Introduction

Over the last two decades, immunotherapy has come to the forefront of cancer treatment. The number of immune modulating agents and modalities being brought to the bedside is rapidly accelerating along with burgeoning activity to identify tumors and patients who would most benefit from these novel approaches. Within the complex immune contexture of cancer, tumor-infiltrating lymphocytes (TIL) may be programmed and produced in the periphery from secondary lymphoid organs (SLO) and potentially access the tumor microenvironment (TME), thereby receiving support by tertiary lymphoid structures (TLS). SLO and TLS are broadly characterized as sites wherein a host immune response can be activated, modulated, and perpetuated. SLO encompass classically known structures, including lymph nodes, Mucosa-Associated Lymphoid Tissue (MALT), and Peyer’s patches. TLS share many structural characteristics to SLO; however, it is their differences in composition, development within the TME, and role in immunotherapy outcomes that are proving critical.

**Figure 1:**
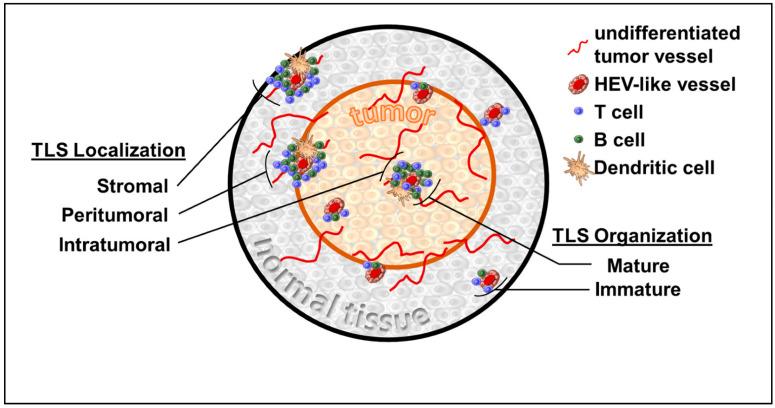
Tertiary lymphoid structures (TLS) variably present within surrounding normal tissue stroma, peritumoral interface, and within tumor. TLS maturation can vary greatly from disorganized clusters of cells to well-developed primary and secondary follicular structures.

TLS are fundamentally non-orthotopic accumulations of variably organized lymphoid tissue arising in the setting of, and as a response to, immune-driven inflammation. While this immune state can be the sequalae of malignancy, other processes, including infection, graft rejection, and autoimmunity, may manifest TLS [1]. TLS appear to lack the formal encapsulation that hallmarks SLO, such as lymph nodes, but share many features of their cellular composition, including stromal cells, antigen presenting cells, high endothelial venules (HEV), and lymphocyte-enriched compartments [2,3] (Figure 1). Specifically, TLS are loosely organized with HEV-like structures that differ from classical HEV found in peripheral lymph nodes. While both express peripheral node addressin (PNAd^+^), the HEV-like structures in TLS are flat compared with the plump cuboidal endothelial cells found in SLO [4]. Similar to SLO, the development of HEV-like structures within TLS appears to be dependent upon lymphotoxin β receptor (LTβR) signaling [4,5]. Preclinically, HEV-like structures associated with TLS were induced via Treg depletion [6], VEGF blockade combined with checkpoint inhibition [7], and via chemokine manipulations of the TME [8]. A variable composition of T and B cells surround these HEV-like structures with particular cellular constituents having been linked to overall survival [9]. Despite these characteristic features, the metrics used to identify TLS within tissue samples is variable. This can lead to the obfuscation of clinical applicability, while also making mechanistic investigations difficult to compare. Much like SLO organogenesis, TLS develop on a spectrum and can be identified at various stages of maturation (i.e., lymphoid aggregates or aggregates with germinal centers). Beyond this structural heterogeneity there are also factors, such as immune cell composition, the presence of HEVs, and numerous gene signatures, that are variably used to identify the presence of TLS. The forthcoming summary of TLS within specific malignancies highlights the importance of recognizing the heterogeneity of TLS as clinically applied thus far.

It is within this immunologic role that TLS appear to not only be associated with patient prognostication, but also in predicting treatment response and possibly even being the target of novel therapeutic strategies clinically.

### 1.1. TLS and Breast Cancer

The role of immune cell infiltration on patient prognosis was extensively queried in breast cancer and indeed studies demonstrate the importance of the microenvironment in determining clinical outcomes. Early work indicated that immune infiltration in breast cancer in the form of increased inflammation was key in the outcomes of breast cancer patients. Histopathologic examination of breast cancer patients who had undergone surgical resection found that diffuse intratumoral inflammation was associated with histologic grade [10]. Those patients who had more advanced cancer interestingly demonstrated that increased intratumoral inflammation was associated with both improved disease-free survival and overall survival. Additionally and more specifically, in a cohort of primarily operated ductal cancer patients, intratumoral lymphocytic infiltration by T-cells was associated with improved survival [11]. When TLS were examined in a selection of patients who underwent upfront surgery, H&E examination revealed that there were intratumoral lymphoid aggregates consistent with TLS, as well as PNAd^+^ HEV that were only noted to be found within the TLS and not in any other areas of examined breast tissue. TLS were specifically found to be present in nearly one-third of that cancer cohort [12]. Additional investigations into breast cancer immune cell infiltration found that tumors with increased infiltrates tended to be characterized by formal organization into TLS and that those structures also retained the majority of *CXCL13*-producing T follicular helper (Tfh) cells and germinal centers. A derivative 8-gene signature, which included detection of the aforementioned *CXCL13* and Tfh, was found to be associated with improved disease-free survival in systemic-adjuvant naïve patients and improved tumor response to those treated with neoadjuvant chemotherapy [13]. In a subpopulation of breast cancer patients who were hormone receptor negative and HER2+ that were then treated with adjuvant Trastuzumab, TIL were more abundant within tumors who had increased TLS and their presence was a useful prognosticator [14].

Interestingly, the role of TLS in the initiation/maintenance of the host anti-tumor milieu could also be related to the structural organization and composition, rather than just the ability of lymphocytes to enter the solid tumor. In a cohort of breast tumors which underwent primary surgical resection, TLS were found to be associated with high lymphocyte infiltration in the whole patient cohort; however, in a subset of patients who were HER2^+^, TLS were associated with improved disease-free survival independent of TIL levels. Most intriguing, this study was conducted in a patient population in the pre-targeted therapy area and almost no patients received trastuzumab [15]. This indicates a particularly germane role for TLS within the tumor-immune response balance; one in which the addition of targeted therapy may be exceedingly beneficial. Thus, the identification of TLS in pre-treatment biopsies and samples could provide important information related to planning; however, given current histopathologic constraints, broad TLS detection may not be feasible in limited tissue specimens. To alleviate the concern for accurate representative tumor biopsies, proxy metrics for the presence of TLS could be applicable. In a cohort of triple negative breast cancer patients, TLS as identified by HEV density (measured by MECA-79) were found to be a reasonable marker for TLS in resected specimens and that tumors with higher HEV density tended to have a higher rate of pathologic response to neoadjuvant chemotherapy and improved disease-free survival. Therefore, this detection of HEV density has meaningful impact on prognosticating patients who are undergoing treatment planning [16]. Although these observations were seen in response to traditional chemotherapy, the role of immune responses associated with chemotherapy are increasingly recognized and may underpin these observations [17,18].

### 1.2. TLS and Colorectal Cancer

Malignancy-instigated immune responses are well described in colorectal cancer and the presence of TIL and specific TIL subpopulations can be important prognosticators. Initial studies demonstrated that the location of immune cell infiltration and specific cellular populations could prognosticate patients even beyond traditional metrics [19]. Indeed, both TIL and specific T-cells, including CD3^+^, CD8^+^, CD45R0^+^, and FOXP3^+^, were noted to be important contributors to patient prognostication [20]. When more organized TIL in the form of TLS were examined in colorectal cancer, some interesting patterns emerged.

In resected specimens of colorectal adenocarcinoma, marked peri-tumoral TLS were identified in a particularly distinct stage of organ maturation. A derivative gene signature that encompassed the expression of TLS determinant chemokines (*CCL2*, *CCL3*, *CCL4*, *CCL5*, *CCL8*, *CCL18*, *CCL19*, *CCL21*, *CXCL9*, *CXCL10*, *CXCL11*, and *CXCL13*) correlated with the presence of TLS and in a highly selected subgroup with improved patient prognosis and overall survival [21]. In another study which focused on locoregionally advanced tumors which had undergone resection, TLS were found to be present at the invasive front of tumors [22]. Some of these aggregates were previously characterized as Crohn-like reactions [23], but in this more contemporary examination, these structures demonstrated expression of key chemokines, including *CXCL13* and *CCL21,* as well as the presence of PNAd^+^ HEV. In fact, the HEV characterized were only noted to be localized to these lymphoid aggregates and not with other tumoral or non-tumoral tissue, lending credence to their role in TLS function. Importantly, in a subset analysis, patients who had earlier disease (stage II) versus later disease (stage III) significantly correlated with disease-free interval indicating that there may be a time during which TLS play a crucial role in immune-mediated tumor control, but that after a certain threshold, immune escape renders them ineffective (at least in the localized disease population) [22]. This led to the notion that TIL and TLS may play an exceedingly relevant role in non-metastatic colorectal disease, outperforming traditional disease recurrence prediction metrics [24]. Their significance may even carry over to the metastatic setting. In a study which examined colorectal metastasis to the liver, TLS were found in a majority of patients who had undergone liver resection metastasectomy, regardless of systemic chemotherapy exposure [25]. The TLS were noted to be localized to the tumor–parenchyma interface and, additionally, could not be identified within normal liver tissue. The density of TLS in these patients subsequently prognosticated patient outcome, with those patients with increased TLS having improved recurrence-free survival independent of prior systemic chemotherapy exposure. Given this seeming contradiction in the role of TLS in non-metastatic, but locoregionally advanced disease and metastatic disease, there may be an immunologic niche in which TLS are an important keystone in both newly established local disease and newly developing metastasis (i.e., early phases of tumor growth before immune escape occurs).

### 1.3. TLS and Hepatocellular Carcinoma

While a preponderance of data across tumor types characterizes TLS as key in the antitumoral coordination of the host immune system, investigations in hepatocellular carcinoma (HCC) provide insight into the mechanisms of such responses. The location of TLS in relation to the tumor appears to be extraordinarily important in their ability to incite effective immune-regulated tumor control. Indeed in non-cancerous liver parenchyma, the presence of TLS allowed for a local microniche wherein malignant progenitor cells could appear and eventually progress into tumors [26]. These patients with high TLS density in non-cancer parenchyma had increased late disease recurrence (>2 years) following primary tumor resection. Moreover, a chemokine gene-expression score derived from these patients, which measured *CCL18*, *19* and *21*, *CXCL9-11* and *13* and *CCL 2*, *3*, *5* and *8*, was an accurate prognosticator for HCC patients, independent of traditional clinical factors. Given that HCC tends to arise in the setting of chronically inflamed parenchyma, the role of TLS in tumorigenesis in this setting is distinct from that which was seen in other tumor types. As opposed to peritumoral TLS, intratumoral TLS appear to play a different role in tumor progression and prognosis [27]. In a cohort of patients who had undergone primary resection, intratumoral TLS were present in nearly half of patients. TLS presence did not appear to correlate with clinical variables that would indicate parenchymal inflammation, such as alcohol consumption or viral hepatitides. Multivariate analysis revealed that TLS correlated with an improved risk of early recurrence. A gene-expression signature that represented TLS presence was also shown to be associated with a lower risk of early recurrence. Perhaps most interestingly, tumors which had TLS presence also tended to be PD-1 high, and with the emerging data regarding the application of checkpoint blockade in the management of HCC, TLS may prove to be key in this treatment avenue [28].

### 1.4. TLS and Lung Cancer

Within lung cancer, the presence of TLS was identified as a progression of lymphoid aggregates that previously was only described in neonatal tissue. In this setting, it was termed bronchus associated lymphoid tissues (BALT) and these were known to involute by adulthood [29]. However, examination of early stage, resected non-small cell lung cancer (NSCLC) revealed the presence of TLS (termed tumor-induced BALT). These aggregates were characteristically aligned with TLS as described in other tumor types, including the organized presence of T- and B-cell populations and lysosome-associated membrane protein positive dendritic cells (LAMP^+^ DC). Interestingly, TLS had no discernable presence within normal lung parenchyma distant from the tumor. Additionally, when intratumoral TLS detected by LAMP^+^ DC were present within these early-stage resected specimens, there was a univariate correlation to improved disease-free survival, disease-specific survival, and overall survival [30]. TLS presence did not appear to vary by smoker status, raising an important distinction from other tumor types where intratumoral TLS were associated with the presence of tumoral and non-tumoral tissue inflammation.

In NSCLC, intratumoral TLS may also support the production of memory/effector cells that remain within the host and continue to provide immune-derived tumor control even after the offending nidus had been resected. This theoretical mechanism is further bolstered by a follow-up examination of resected NSCLC patients, including those who were treatment naïve and those that received systemic neoadjuvant therapy [31]. Histopathologic examination not only revealed the expected cellular subpopulations, but also the presence of B-cell populations in variable stages of differentiation, indicating that this may be the mechanistic underlay for a humoral response. Indeed, additional evaluation revealed that these antibody-secreting plasma cells were capable of recognizing known tumor antigens expressed by neoplastic cells. Additionally, the density of B cells present within the TLS correlated with increased median survival regardless of treatment exposure, reinforcing the role that the adaptive immune response plays in sustaining tumor control [31].

### 1.5. TLS and Urothelial Cancer

Urothelial bladder cancer has a long-standing connection with immunology, beginning with the treatment of malignancy with Bacilus Calmette-Guerin (BCG). Additionally, there was a focus on the immune infiltration patterns of bladder cancers, especially given the recognition that low grade variants and high grade variants may be distinct clinical entities with significant impact on patient prognosis. In an early study examining immune cell infiltration in a cohort of both high grade (muscle-invasive bladder cancer, MIBC) and low grade bladder cancer (non-muscle-invasive bladder cancer, NMIBC), TLS were only present in 25% of NMIBC cases, while they were present in 75% of MIBC cases [32]. Moreover, although specific patient outcomes were not examined in this study, the relationship of TLS and prognosis may be more nuanced. In a study of patients with locoregionally advanced urothelial cancer who underwent treatment with ipilimumab and nivolumab, pretreatment samples revealed that clinical non-responders tended toward enrichment of immature TLS more than responders [33]. Alternatively, another trial looking at pretreatment samples of urothelial carcinoma patients who received neoadjuvant durvalumab (anti PD-L1) and tremelimumab (anti CTLA-4) found that responders tended to have higher TLS [34]. In an exploration of these dialectic findings, data indicate that the specific cellular composition of TLS (defined as distinct clusters) differed among immunotherapy responders versus non-responders (FoxP3 T-cell low TLS). In addition, post treatment samples demonstrated cluster changes based on exposure to immunoactive agents (more TLS that were macrophage low in treated versus untreated) [35].

### 1.6. TLS and Melanoma

TLS in melanoma were originally described in metastatic disease where samples of cutaneous progression demonstrated ectopic lymphoid aggregation, as well as the presence of PNAd^+^ HEV. This early data also attempted to identify similar aggregates in primary lesions using follicular DC expression (defined by CD21L) as a molecular proxy for TLS, and found no presence [36]. However, in a larger series of examination, TLS were identified within primary melanoma, including the presence of mature DCs, B- and T-cell clusters, and MECA-79^+^ HEVs [37]. Additionally, the presence of these TLS may be strong determinants of patient outcome [37,38].

In a study of patients undergoing melanoma metastasectomy, TLS were identified in 47% of the samples and were associated with a lower risk of recurrence and improved overall survival following resection [9]. Furthermore, the TLS that were observed varied greatly in their maturation state and it appeared that the B-cell population associated with the TLS influenced clinical outcomes.

The study of HEV within melanoma also provided an important vantage of the interplay between the immune system and melanoma tumor control. In a study of primary resected melanoma, MECA-79^+^ HEV was identified in 68% of tumors, but not seen at all in distant, normal cutaneous samples [39]. Additionally, the density of HEV appeared to vary by histologic type, consistent with subsequent studies [40]. HEV density also correlated with histopathologic tumor regression, as well as TIL densities (CD20^+^, CD3^+^ and CD8^+^). Specific chemokine signatures associated with antitumor T-helper activity correlated with HEV density, while anti-tumor/tumor escape T-regulatory cell density did not appear to be associated. Taken in aggregate, this provides indirect evidence of the role of HEVs in immune-system priming driven by TLS.

### 1.7. TLS and Immunotherapy

The presence of TLS appears to be correlated with patient prognosis in variable tumor types. While the aforementioned work in breast cancer indicates that TLS may be useful prognosticators in the setting of traditional chemotherapy, responses to novel immunotherapies may be more relevant given the important immunologic niche that TLS may serve.

In one study of resected melanoma specimens, TLS were identified and T-cell subpopulations (CD8^+^) were noted to be localized to just outside the TLS, indicating that the TLS may be a focus of activation for the systemic immune response to the tumor [41]. Patient prognosis in this study reinforced this idea in which patients who had either CD8^+^ infiltration or TLS presence had improved survival; those with both had the best survival; and those with neither had the worst. Additionally, this study was able to derive a gene signature using key determinants of TLS formation in melanoma and tumors were subsequently able to be stratified as TLS high, intermediate, and low. This signature was then applied to the biopsy specimens of patients who went on to receive CTLA4 blockade. TLS-high signature patients demonstrated a significantly improved overall survival in a stepwise fashion compared with those with TLS intermediate or TLS low signatures after treatment with CTLA4 blockade. This finding was reciprocated in an additional cohort of melanoma patients and highlights the importance of TLS in modulating the immune response to tumors and allowing the more efficacious application of checkpoint blockade.

In advanced bladder cancer, a transcriptomic investigation of the B-cell migration chemokine *CXCL13* revealed that it may be a useful marker for the presence of TLS. Furthermore, it appeared to correlate with improved tumor response and patient survival and was present only in patients who were treated with an anti-PD-L1 monoclonal antibody [42].

Multiomic data indicate that TLS, and certain cellular subtypes within these lymphoid aggregates, may play an important role in the tumor responses of melanoma and renal cell carcinoma to immune checkpoint blockade [43]. In a study utilizing clinically annotated transcriptomic data, a 12-chemokine based gene signature for TLS was developed and applied to a subset of patients with cutaneous melanoma and NSCLC [44]. The patients included failed first line treatment but were immunotherapy naïve and were then treated with anti-PD-1 agents. Patients who responded to treatment had higher tumor TLS signatures and those with high TLS signatures had improved overall survival. A similar trend was seen in another cohort of cutaneous melanoma patients treated with anti-CTLA-4 agents.

In soft tissue sarcoma, tumors were able to be categorized based on immune microenvironment composition and those malignancies with a classification characterized by TLS showed both improved survival and a better response rate to PD1 blockade-based therapy [45].

The role of TLS in the immunomodulatory control of tumor progression may even supersede more traditional metrics. In one study, a variety of pre-immunotherapy tumor samples, including colorectal, breast, sarcoma, and NSCLC, were examined. Patients with mature TLS appeared to derive the most benefit from checkpoint blockade, with improved objective tumor responses. Additionally, these TLS were more prominent amongst long term survivors and patients whose tumors demonstrated TLS also had improved progression-free survival. Most interestingly, these responses to checkpoint blockade, as evidenced by immunotherapy efficacy, were independent of both CD8^+^ T-cell infiltration and patient PD-L1 status [46].

### 1.8. TLS Organogenesis and Inducible TLS

SLO are the product of a highly coordinated prenatal organogenesis that allows for antigen sampling, exposure to innate immune cells and the consequent expansion, differentiation and dissemination of naïve T and B cells now exposed to these defined antigens. TLS allow for a similarly refined process but in the post-natal/organogenesis setting [47].

TLS share many of the structural foundations of SLO, including (1) the presence of HEV-like structures, (2) distinct cell clusters characterized by T-cell rich areas, LAMP^+^ DC clusters, and B-cell enriched areas organized around germinal centers, and (3) the secretion of chemotactic cytokines (i.e., *CXCL13*, *CXCL12*, *CXCL10*, *CCL21*, and *CCL19*) [2].

The crux of TLS functionality may lie in the presence of HEV-like structures. Immune surveillance is dependent on the ability of lymphocytes to transition to and from the blood. This circulation allows relatively rare naïve, antigen-specific lymphocytes to broadly surveil the host organism. This movement is dependent on specialized blood vessels, HEVs, which were well characterized in relation to peripheral lymph node function [48] but were more recently implicated in TLS function, as well as in the form of HEV-like vessels [49,50]. In vivo data appear to reiterate the importance of HEV function. In one murine examination, sarcoma tumors which were immune-edited for surveillance escape demonstrated less MECA-79^+^ HEV. Subsequently, the number of HEV-associated endothelial cells (HEC), as well as specific TIL, was noted to increase when treated with combined CTLA-4 and PD-1 blockade. When a sample of pre-treatment metastatic lesions was examined, the presence of HEV was higher in patients who responded to combined CTLA-4 and PD-1 blockade and this response was likely a driver for the observed improvement in both progression-free survival and overall survival seen with those that had been treated with combined therapy [51].

Collectively, TLS may provide the immune-activating functionality of SLO, while providing distinct benefits that foster antitumor activity outlined succinctly by Schumacher and Thommen: (1) **Speed**—TLS may allow for rapid lymphocyte priming by circumventing the extraneous trafficking needed for SLO function; (2) **Efficiency**—localized lymphoid contexture is fostered by the approximation of lymphocyte and recognized tumor-associated antigens; (3) **Control**—TLS allow for immune response refinement by aggregating immune cells and malignant targets; and (4) **Survival**—lymphocyte homeostasis is modulated by TLS-cell population factors [52]. Indeed, these theoretical advantages appear to be borne out when the presence of TLS is correlated with improved prognosis in specific malignancies [53].

**Figure 2:**
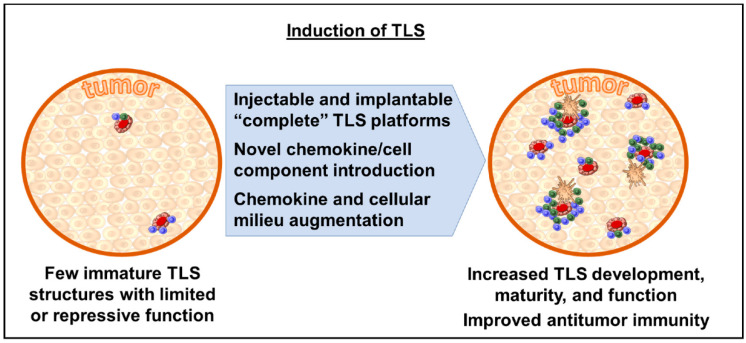
Methods of inducing TLS within tumor microenvironment.

Consequently, the ability to generate inducible TLS (“iTLS”) presents a potentially attractive avenue for therapeutic intervention, whereby TLS could be introduced into the tumor microenvironment and allow the host immune system to either intuitively prime itself to an antitumor state or allow for the more efficacious application of immunotherapy agents, such as checkpoint blockade (Figure 2). One such proposed preclinical model includes an injectable/implantable platform which contains a cellular scaffold, engineered cell populations, and integral chemokines as a self-contained package [54]. Another approach may include the introduction or augmentation of host chemokines/cellular components to allow for de novo organogenesis; however, this may be more theoretical for the present, as the exact sequence of factor expression, lymphocyte migration, and structure maturation is yet to be fully elucidated [55]. More recent work is generating new insight into the mechanistic underpinnings of the cellular differentiation cascade leading to TLS formation and could be the cornerstone for future micro-environment augmentation, making checkpoint blockade more efficacious [56].

## 2. Conclusions

As the role of the tumor microenvironment in regulating patient prognosis and therapeutic response is now more evident, immune augmentation is quickly becoming the first line approach for tumor control and even eradication. TLS are unique, immunologically advantageous anatomic structures that allow for improved patient prognostication and, perhaps most importantly, are potentially an underutilized avenue for therapeutic intervention.
